# Diabetic ketoacidosis complicated by the use of ecstasy: a case report

**DOI:** 10.1186/1752-1947-4-240

**Published:** 2010-08-03

**Authors:** Mirnaluci Paulino Ribeiro Gama, Bárbara Vicente de Souza, Ana Carolina Ossowski, Rafaela Cristina Perraro

**Affiliations:** 1 Alameda Augusto Stellfeld, 2134, CEP:80730-150 Curitiba-PR, Brasil; 2 Rua Eusébio de Queirós, 287; AP 403, CEP:89203-100 Joinville-SC, Brasil; 3 General Aristides Athaíde Jr, 602; AP 301, CEP:80730-370 Curitiba-PR, Brasil; 4 Rosa Kaint Nadolny, 225 ap 1802, CEP:81200-290 Curitiba-PR, Brasil

## Abstract

**Introduction:**

Ecstasy (3,4-methylenedioxymethamphetamin), a hallucinogenic amphetamine, is often used by young people, especially at 'raves'. This illicit drug can cause many metabolic changes and its use, when associated with prolonged exercise, may exacerbate ketoacidosis in type 1 diabetic patients.

**Case presentation:**

This is a case of ketoacidosis complicated by the use of ecstasy in a 19-year-old insulin-dependent diabetic Caucasian woman.

**Conclusion:**

The use of ecstasy may trigger diabetic ketoacidosis in patients with a preexisting metabolic disorder

## Introduction

A considerable number of young people are exposed to 3,4-methylenedioxymethamphetamine (MDMA -, also known as 'ecstasy') [1], a synthetic compound with structural and pharmacological characteristics similar to those of amphetamines. The use of this drug has increased considerably over the last 15 years [2]. The drug was originally developed as an appetite suppressant, although it was never promoted commercially for that purpose. Given its psycho-affective properties of inducing euphoria, disinhibition and sexual arousal, the drug was later used as an adjunct to psychotherapy. In the 1980s, MDMA became a trendy drug of abuse, particularly at rave parties and dance clubs. However, its potential for abuse was soon recognized, thus prompting government officials to place restrictions on its use [3].

In Brazil, the first shipments of ecstasy arrived in São Paulo in 1994, mainly from Amsterdam, and began to be trafficked, mostly at raves and dance clubs [4].

Ecstasy is mistakenly seen by young people as a 'safe' drug compared with amphetamines. However, it not only has some of the toxicity of amphetamines but also other detrimental acute effects (including increased risk of fatal arrhythmias, rhabdomyolysis, acute kidney failure, hyponatremia) and chronic effects (such as persistent panic disorder symptoms, depression, insomnia and memory impairment) [5].

We describe ecstasy use in a young woman with type 1 diabetes mellitus (T1DM) with underlying poor control who developed diabetic ketoacidosis.

## Case presentation

A 19-year-old single diabetic Caucasian woman had been on 52 units of NPH insulin (30 units in the morning and 22 at bedtime) and 12 units of regular insulin (four units before meals) daily for six years. She presented at the emergency department with nausea, vomiting and malaise of a two hour duration, after ingesting half a tablet of ecstasy. She reported having danced strenuously and drunk approximately 4L of mineral water after using the drug. Polyuria, polydipsia and altered capillary glycemia values had been present for approximately one month, but were overlooked by the patient. She denied fever, dysuria, coughing or irregularity in the administration of insulin, but had noticed slight urine turbidity over the preceding 15 days. She denied having used ecstasy in association with alcohol, or any other combination, and claimed that this had been her first experience of using any illicit drug. She had been admitted to another hospital three months earlier for signs and symptoms of diabetic ketoacidosis of unknown origin. Her last HbA1 c was 12%. On admission, she presented with acetone breath and was dehydrated, tachycardic, tachypneic, normotensive and confused.

The physical examination was unremarkable, except for a mild diffuse abdominal pain with no signs of peritoneal irritation. Her temperature was 37.3°C and the chest X-ray was normal. Urine analysis showed pronounced ketonuria and an absence of pyuria; a urinary bacterioscopy did not reveal bacteria; arterial blood gas measurement revealed clinically important metabolic acidosis (Table 1); and lactate was in the normal reference range.

**Table 1:** Laboratory tests

Tests	Admission	Discharge
Hematocrit/hemoglobin	42.5%/13.7 g/dL	35.9%/12.6 g/dL

White blood cells	23.100/mm^3^	4.460/mm^3^

Neutrophils	18.480/mm^3^	2.720/mm^3^

Sodium	138 mmol/L	137 mmol/L

Potassium	5.4 mmol/L	3.3 mmol/L

Serum glucose	540 mg/dL	124 mg/dL

Urea/creatinine	42/1.2 mg/dL	23/0.82 mg/dL

Gasometry		
pH	7. 123	7. 535
Bicarbonate	2.5 mmol/L	20.7 mmol/L

Chloride	110 mEq/L	98 mEq/L

Anion gap	30.9	21.6

The patient received immediate intravenous rehydration, regular insulin using a continuous infusion pump and potassium replacement. She progressed with marked improvement in the symtomatology after 24 hours. She was discharged from hospital three days after diagnosis and prescribed 48 units of NPH insulin daily.

## Discussion

Ecstasy is one of the few drugs with dual classification: it falls among the stimulants and the hallucinogens and is sometimes classified as a hallucinogenic amphetamine [6]. The drug acts primarily by promoting a massive release of serotonin from the presynaptic cleft and, additionally, inhibits serotonin reuptake and increases its levels in the postsynaptic receptors. The drug is also a potent dopamine and noradrenaline releasing agent [7]. Typically, users ingest at least one tablet of ecstasy with doses ranging between 50 mg and 200 mg, although it is often mixed with other substances [8]. The drug is readily absorbed by the gastrointestinal tract, with peak action two to four hours after ingestion and a half-life of approximately nine hours [4].

The acute effects of ecstasy include: a feeling of improvement in interpersonal skills: loquacity; an enhanced perception of music; a reduction in fatigue; a heightened alertness; a sense of increased physical and mental powers; and euphoria. The drug produces physiological alterations such as: hyperthermia; acute sympathomimetic effects (elevated heart rate and arterial blood pressure); exacerbation of anxiety; and activation of the hypothalamic-pituitary-adrenal axis. The most frequently reported side effects are arrhythmias, hyperthermia, kidney failure, seizures and intracranial hemorrhage [9].

It is important to note that ecstasy promotes an increased antidiuretic hormone release, which may lead to symptomatic hyponatremia and hypo-osmolarity. Other factors contributing to hyponatremia include psychogenic polydipsia and volume depletion by loss of free water. Thus, excess water intake could eventually become dangerous, even fatal, since it could lead to seizures, herniation and cerebral edema [10]. The woman in our case had normal serum sodium levels on admission to hospital.

One of the most remarkable acute effects of ecstasy is a pronounced increase in body temperature of up to above 42°C. Hyperthermia can result from the effects of the drug on the central nervous system, prolonged exertion (for example, dancing strenuously during a rave night) and environment conditions (crowds or hot environments). Both the stimulant effect of amphetamines and serotonin syndrome can contribute to severe hyperthermia which may cause dehydration, disseminated intravascular coagulation, seizures and even rhabdomyolysis [3]. A large number of ecstasy deaths are thought to be directly or indirectly related to the effects of hyperthermia.

The increase in body temperature, in conjunction with the strenuous exercise of vigorous dancing, makes drinking water a necessity for the ecstasy user [4]. Although our patient had taken a small dose of the drug along with the compulsive intake of 4L of water throughout the night, she had difficulty in achieving full water-electrolyte replacement which was evident in her case as she was severely dehydrated. This, in conjunction with hyperglycemia, metabolic acidosis and ketonuria, was strongly suggestive of diabetic ketoacidosis.

Seymor and colleagues reported two cases of diabetic ketoacidosis complicated by ecstasy use. Both were insulin-dependent diabetics and ingested ecstasy during a rave party, during which they danced strenuously. They showed good clinical evolution after fluid replacement and the administration of insulin, although one of the patients developed bronchopneumonia and needed intubation and mechanical ventilation [11].

Lee and colleagues conducted a study with patients who had type-1 diabetes mellitus, assessing the impact of substances of abuse on the level of acidosis of the diabetic ketoacidosis. Of the 19 patients evaluated, six had used ecstasy. Drug users were found to develop more severe acidosis than non-users of illicit substances [12].

A study by De Micheli and colleagues had a sample of 6417 students and showed that the prevalence of young people who have already tried drugs of abuse is high in secondary schools in the interior of the state of São Paulo; only 0.9% had already consumed ecstasy. The average age at the time of the first experience with ecstasy is 20 years - ranging between 13 and 41 years. The pattern of ecstasy use in Brazil comprises young men who are: polydrug users; heterosexual; single; have a college degree or some college education; and belong to the upper socioeconomic classes. This is a drug use pattern similar to that seen in other countries [13].

A survey conducted with Brazilian users found that 'a concern with physical health' is the most influential factor in the frequency of ecstasy use. However, only a minority of users wanted to reduce the use or discontinue using the drug [14].

The association between decompensated diabetes and the use of illicit drugs is frequent. Some authors see poor control of diabetes as a possible warning sign for the likelihood of a young person becoming involved with illicit drugs. Adolescents are most vulnerable to drug abuse and often ignore warnings about the danger of using such drugs means there is an increased risk of death among this age group [10]. This risk should, therefore, be addressed in all medical interviews with adolescents.

## Conclusion

The use of ecstasy may trigger diabetic ketoacidosis in patients with a preexisting metabolic disorder. This is demonstrated in the present case and other cases described in the published literature. The strenuous exertion often associated with the use of this drug may compound the problem. Often patients do not wish to discontinue ecstasy use, despite their being made aware of the potential risk of this drug. The woman in our case was not aware of the side effects of ecstasy. We believe that improved screening and diagnosis, as well as education on the seriousness of the use of illicit drugs, are needed both by all young people but especially those who are prone to developing metabolic disorders, such as diabetics.

## Abbreviations

MDMA: 3,4,-methylenedioxymethamphetamine; T1DM: type 1 diabetes mellitus.

## Consent

Written informed consent was obtained from the patient for publication of this case report. A copy of the written consent is available for review by the Editor-in-Chief of this journal.

## Competing interests

The authors declare that they have no competing interests.

## Authors' contributions

ACO, BVS and RCP analyzed and interpreted the patient data, reviewed the literature and wrote the first draft of the report. MPRG was the chief clinician, edited the first draft of the manuscript and assisted in the literature review. All authors read and approved the final manuscript.
